# Self-Assembly Dipeptide Hydrogel: The Structures and Properties

**DOI:** 10.3389/fchem.2021.739791

**Published:** 2021-09-01

**Authors:** Liangchun Li, Li Xie, Renlin Zheng, Rongqin Sun

**Affiliations:** ^1^ School of Life Science and Engineering, Southwest University of Science and Technology, Mianyang, China; ^2^ School of Materials Science and Engineering, Southwest University of Science and Technology, Mianyang, China

**Keywords:** hydrogel, dipeptide, self-assembly, ultrashort peptide, biocompatible

## Abstract

Self-assembly peptide-based hydrogels are well known and popular in biomedical applications due to the fact that they are readily controllable and have biocompatibility properties. A dipeptide is the shortest self-assembling motif of peptides. Due to its small size and simple synthesis method, dipeptide can provide a simple and easy-to-use method to study the mechanism of peptides’ self-assembly. This review describes the design and structures of self-assembly linear dipeptide hydrogels. The strategies for preparing the new generation of linear dipeptide hydrogels can be divided into three categories based on the modification site of dipeptide: 1) COOH-terminal and N-terminal modified dipeptide, 2) C-terminal modified dipeptide, and 3) uncapped dipeptide. With a deeper understanding of the relationship between the structures and properties of dipeptides, we believe that dipeptide hydrogels have great potential application in preparing minimal biocompatible materials.

## Introduction

Hydrogels are cross-linked by three-dimensional networks of modified molecules that hold a large amount of water, which were first reported by Wichterle and Lím (1960). By definition, water must account for at least 10% of the total weight (or volume) of hydrogels, which is very similar to natural tissue. Due to the high water content, favorable structural features, and biocompatibility, hydrogels have great potential in biomedical applications such as drug delivery, tissue engineering, sensing, and cell culture scaffolds (Ahmed, 2015; Ghosal et al., 2018; Zou et al., 2020). Based on the different gelation mechanisms, hydrogels can be classified into chemical hydrogels and physical hydrogels (Mahinroosta et al., 2018). The chemical hydrogels are cross-linked *via* covalent bonds, resulting in high mechanical strength, structural stability, shape memory, and irreversible properties (Liyan et al., 2018). The irreversible properties deprive them of self-healing and impair their injectability (Tu et al., 2019). More importantly, those limit cell proliferation and migration and hence limited application in three-dimensional cell culture (Wang et al., 2019, 2020). Physical hydrogels, also known as supramolecular hydrogels, could be produced by a molecular self-assembly process (Li et al., 2020; Xian and Webber, 2020; Li et al., 2021b). Molecular self-assembly relies on noncovalent interactions, such as hydrogen bonding, hydrophobic interactions, aromatic π–π stacking interactions, and electrostatic interactions, which are weak and reversible (Whitesides and Boncheva, 2002; Webber and Pashuck, 2021). The dynamic reversible nature of these interactions endows them with self-healing and shear thinning properties; hence, they are particularly suitable for biomedical applications, as they are more flexible and can be easily injected (Hu X. et al., 2020; Gupta et al., 2020; Vazquez-Gonzalez and Willner, 2020).

The physical hydrogelators include natural polymers, such as polysaccharides (Dai et al., 2019; Chen et al., 2020; Lei et al., 2020), proteins (Wlodarczyk-Biegun et al., 2016; Fisher et al., 2017; Singh et al., 2017; Zhao et al., 2018), and polynucleotides (RNA and DNA) (Korah et al., 2018; Li et al., 2019b), and synthetic materials, such as poly (lactic-co-glycolic acid (PLGA) (Ko and Kwon, 2020), polyglycolic acid (PGA) (Prabhu et al., 2020), and polylactic acid (PLA) (Basu et al., 2016). These various polymers establish complex 3D networks of intermolecular interactions between building blocks to form macroscopic objects. Besides polymers, low-molecular-weight gelators (LMWGs) (generally <2000 Da) are of great interest because the associated gelators have intrinsic self-assembly ability to form different morphologies of fibers, rods, ribbons, and nanotubes, which could be used to build attractive tools for various biomedical applications on account of their high biocompatibility and low toxicity (Nolan et al., 2017; Ramin et al., 2017; Piras et al., 2021). Among the LMWGs, self-assembly peptide-based hydrogels are well known and have been the subject of many studies because the peptides are the most attractive building blocks, which can be readily controlled by changing the amino acid sequence of the peptides and by modifying the side chains of the peptides (Frick et al., 2017; Fu et al., 2021; Zhang et al., 2021). In particular, many small amino acid sequences responsible for the biological assembly proteins such as the amyloid-beta polypeptide (Aβ42) (Qiu et al., 2015), tau proteins (Pîr et al., 2017), and islet amyloid polypeptide (IAPP) (Fawver et al., 2014) indicated in Alzheimer’s disease, have been identified; hence, researchers can design self-assembly peptides through a biomimetic approach. The shortest self-assembling motif of peptides was dipeptide, for example, L-Phe-L-Phe, a dipeptide from Aβ42, which is also the most widely studied scaffold of supramolecular hydrogelators (Diaferia et al., 2019b). However, despite the easy synthesis and decoration of dipeptide, the prediction and design of dipeptide-based hydrogels are still challenging due to the complex nature of the molecular structure and hydrogel behavior (Fichman and Gazit, 2014; Frederix et al., 2015; Fuentes-Caparrós et al., 2019). Researchers have tried many strategies in dipeptide design to resolve this problem, such as the introduction of a combinational approach, which generated a structurally diverse hydrogel library with more than 2,000 peptides (Li et al., 2019a).

Recently, self-assembly hydrogels based on the longer (Levin et al., 2020; Mondal et al., 2020) or short peptides (Fleming and Ulijn, 2014; Tao et al., 2016; Guyon et al., 2018) and their related gel-based biomedical applications (Fukunaga et al., 2019; Ni and Zhuo, 2019) have been reviewed but in a very limited manner. In this review, we focus on the dipeptide derived from common amino acids that form hydrogels and the possible relationship between the structures and hydrogel behavior. We hope that with a deep understanding of the relationship between structures and properties of dipeptides, the dipeptide hydrogels would have an effective application in preparing minimal biocompatible materials.

## The Structures of Self-Assembly Dipeptide Hydrogelators

**FIGURE 1:**
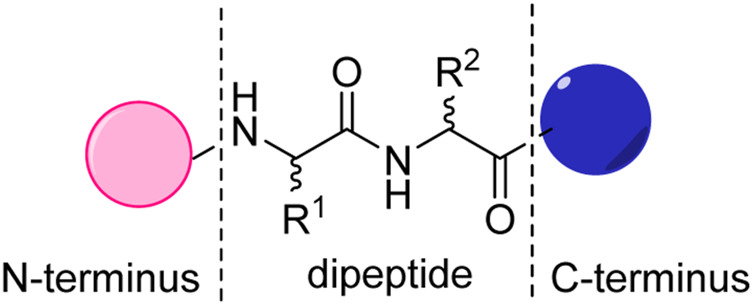
The general structure of dipeptide hydrogelator.

To form a hydrogel, the dipeptide hydrogelator first self-assembled into some kind of one-dimensional (1D) supramolecular structures, like nanofibers and twisted nanoribbons, and then into three-dimensional (3D) networks to ensnare a great number of water molecules inside (Du et al., 2015). This demands that the structure of the dipeptide hydrogelator should greatly facilitate the formation of one-dimensional assemblies. The classic dipeptide hydrogelator is divided into three parts, as shown in Figure 1. The two same or different amino acids are linked *via* an amide bond to form the main chain of dipeptide hydrogelator, which can provide intermolecular hydrogen bonding in the self-assembling process. The other two parts are also important and are recognized as the side chain, which helps the gelator to self-assemble into associated 3D networks. As we know, hydrogel is one of the most typical amphiphilic materials in which solvent molecules are entrapped within the 3D networks under suitable conditions (Bahram et al., 2016). Unlike in covalently connected polymers (polypeptide or proteins), the structure of dipeptide hydrogelator is relatively simple and allows one to tune the formation easily and subtle changes can be applied to the structure that may lead to various hydrogel behaviors: gel or not gel.

The amino acids have different side chains for various hydrophilic and hydrophobic properties. There are the 20 different most common amino acids in nature and each of them has specific chemical characteristics, which have a unique role in protein structure and function. Based on the characteristics of the side chain when it is in contact with water, amino acids can be classified into three categories: hydrophobic (low propensity to be in contact with water), polar, and charged (energetically favorable contacts with water). Due to the simple synthesis of dipeptides, they have significant advantages in regulating physicochemical behavior by peptide sequence design (Bak et al., 2015). Even if there is only one amino acid difference, the self-assembled nanostructures and the resulting hydrogels can be significantly changed (Habibi et al., 2016; Zhou et al., 2019).

The side chains of the N-terminus and C-terminus generally provide an auxiliary function for a better equilibrium of solvability and self-assembly capacity, such as enhancing the hydrophobicity and the intermolecular forces. It has been proven that an efficient way to construct a supramolecular hydrogelator is to attach a π-conjugated group to a short peptide (Adams and Topham, 2010). For example, the Fmoc-Phe-Phe (Fmoc-FF) dipeptide, derived from the β-amyloid peptide, is mediated by a potent combination of hydrophobic, π–π, and hydrogen bonding interactions that result in the formation of hydrogel (Ryan and Nilsson, 2012; Rajbhandary and Nilsson, 2017).

The strategies for preparing the new generation of dipeptide hydrogels can be divided into four categories based on the modification site of dipeptide: 1) N-terminal modified dipeptide; 2) C-terminal modified dipeptide; 3) uncapped dipeptide; 4) cyclic dipeptide. This review will focus on the linear dipeptide (1–3) and other recent reviews about cyclic dipeptide can be found in articles by Yu et al. (2020) and Balachandra et al. (2021).

## COOH-Terminal and N-Terminal Modified Dipeptide

To form an efficient dipeptide hydrogel, it is possible to modify a large aromatic group on the N-terminal of the dipeptide hydrogelator (Fichman and Gazit, 2014). Aromatic moieties can facilitate the self-assembly of peptides and stability of conformations and functions due to the favorite contribution of the aromatic group, such as π–π stacking and hydrophobic interactions. The aromatic group includes fluorenylmethoxycarbonyl (Fmoc), naphthalene (Nap) derivatives, phenothiazine (PTZ), carboxybenzyl (Cbz), azobenzene (Azo), and pyrene (Pyr). The literature has reported that unmodified dipeptides without aromatic capping do not form hydrogels, whereas several N-terminal modified peptides have been used as efficient hydrogelators; the detailed contents are discussed as follows.

### Aromatic-Modified Dipeptide

The Fmoc group is widely used as a protecting group in peptide synthesis; thus, the Fmoc-modified dipeptide hydrogelator is readily studied. In 1995, the first Fmoc-modified dipeptide hydrogelator Fmoc-Leu-Asp was reported by the Vegners group, which forms hydrogel at 2 mg/ml in PBS and was used as a carrier for antigen presentation (Vegners et al., 1995). Following this study, several Fmoc-dipeptides as efficient hydrogelators have been reported, and mostly the Fmoc-dipeptides self-assembled into a fibrous structure in gel state (Tang et al., 2011; Sasselli et al., 2016).

Fmoc-Phe-Phe, an efficient hydrogelator under physiological conditions, has attracted particular interest in contrast to the other dipeptides. Since 2006, this hydrogelator has been well characterized and widely examined for various applications. Jayawarna and coworkers have discovered the gelation properties of seven Fmoc-dipeptides made up of the combinations of the four amino acids: glycine, alanine, leucine, and phenylalanine (Jayawarna et al., 2006). Fmoc-Phe-Phe formed a hydrogel under a pH less than 8, the other five dipeptides Fmoc–Gly–Gly, Fmoc–Ala–Gly, Fmoc–Ala–Ala, Fmoc–Leu–Gly, and Fmoc–Phe–Gly could form hydrogels under the condition of pH < 4, whereas Fmoc–Gly–Phe gave crystals and did not form a gel under any of the conditions tested. Thus, the nature and architecture of the amino acid building blocks could dictate the properties of the hydrogels.

Smith and coworkers have confirmed that the Fmoc-Phe-Phe self-assembled into nanocylindrical fibrils based on π-π interlocked antiparallel β-sheets (Smith et al., 2008). Moreover, they reported that the self-assembly process could result in two apparent pKa values: the first located at pH 9.5–10.2 corresponding to the self-assembly of the dipeptide into paired fibrils consisting of antiparallel β-sheets and the second located at pH 6.2–9.5 related to forming large rigid ribbons by lateral aggregation of fibrils (Tang et al., 2009).

Adams and coworkers designed a series of orthogonal experiments to study the mechanical properties of Fmoc-Phe-Phe hydrogel prepared using different conditions (Raeburn et al., 2012). They have demonstrated that the final pH of the gels, no matter the gel formation method, is the principal determinant for mechanical properties. Moreover, they revealed that the other experimental factors, such as the ratio of organic solvent to water and the nature of the buffers, affect the rheological properties to a lesser extent. Tirelli et al. have also shown that the different homogenization techniques, such as vortex vs. manual or orbital agitation, could dramatically influence the mechanical properties and the self-assembling structures (Helen et al., 2011). All these interesting studies have suggested that the self-assembly process and its inherent mechanism of Fmoc-modified peptides are significantly complicated, and the influencing factors, such as the molecular structures, solvent compositions (including solvent type, polarity, the ratio of organic solvent to water, and the buffer components), solution conditions (pH, ionic strength, ionic type, and valence state, etc.), and external conditions (temperature, homogenization method, photo stimulus, etc.) should be thoroughly clarified.

Another extensively studied Fmoc-based dipeptide is Fmoc-Tyr-Leu (Bai et al., 2014; Fleming et al., 2014). Due to the phenolic hydroxyl of tyrosine, anions can affect the hydrogelation of Fmoc-Tyr-Leu, leading to the transformation from fibrous structures to spherical aggregation. Ultrasonication can also control the self-assembly process of Fmoc-Tyr-Leu by transforming the coiled nanofibers to form spherical aggregates, which means the stacking interactions of the fluorenyl rings are enhanced. Bai group has recently reported two oppositely charged dipeptide Fmoc-Tyr-Asp and Fmoc-Tyr-Lys, which could self-assemble into β-sheet amyloid structures and form tunable hydrogel (Jian et al., 2019). The mechanical and biodegradation properties of the hydrogels could be adjusted by changing the concentration and composition of the dipeptide. They have also demonstrated that the formed hydrogels could form, grow, and naturally release HepaRG spheroids with sizes up to 1 mm, which might be suitable to use as bioinks.

**FIGURE 2:**
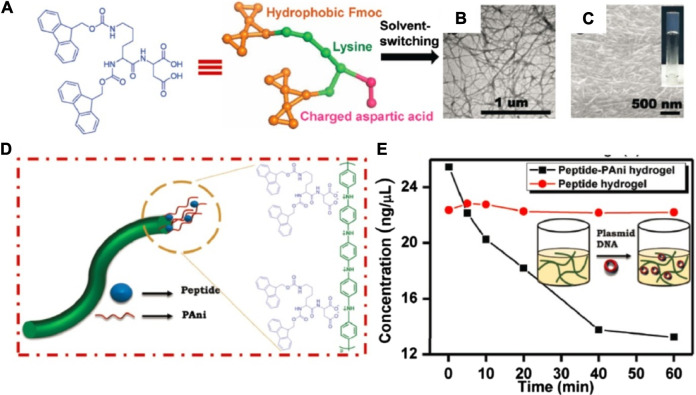
**(A)** The structure of Fmoc-Lys (Fmoc)-Asp; **(B, C)** TEM and HRSEM images of Fmoc-Lys (Fmoc)-Asp hydrogels; **(D)** illustration of the probable structure of the peptide–polyaniline (PAni) formed fibers; **(E)** DNA binding by the composite peptide–PAni hydrogel (Chakraborty et al., 2020).

Over the years, as more and more Fmoc-modified dipeptide hydrogelators were reported, such as Fmoc-Phe-Tyr (Wang et al., 2008; Sadownik et al., 2011; Hughes et al., 2013), Fmoc-Phe-Leu (Ren et al., 2020), Fmoc-Phe-Cys (Wang and Chau, 2012), Fmoc-Phe-Val (Najafi et al., 2021), Fmoc-Tyr-Ala (Bai et al., 2014; Ren et al., 2020), Fmoc-Tyr-Ser (Hughes et al., 2012, 2013; Bai et al., 2014), Fmoc-Tyr-Thr (Hughes et al., 2012, 2013), Fmoc-Tyr-Asn (Hughes et al., 2013), and Fmoc-Gly-Ser, which also self-assembled into fibrillar structures like Fmoc-Phe-Phe, the results indicated that the π–π and hydrophobic interactions of intermolecular Fmoc groups are the main driving forces in the self-assembly process of these systems. Moreover, double Fmoc group-containing dipeptide, Fmoc-Lys (Fmoc)-Asp, as an hydrogelator with the lowest CGC ever reported, 0.002 wt% (28.3 × 10^–6^ M), was reported by Gazit and Wei et al. (Chakraborty et al., 2020). The conductive composite gels formed from Fmoc-Lys (Fmoc)-Asp and polyaniline (PAni) were cytocompatible and exhibited excellent DNA binding properties, suggesting its potential application in DNA biochip fabrication (Figure 2). However, the other analogies of the double Fmoc group-containing dipeptides, Fmoc-Lys (Fmoc)-Ala, Fmoc-Lys (Fmoc)-Cys, Fmoc-Lys (Fmoc)-Asn, and Fmoc-Lys (Fmoc)-His formed hydrogels, but with lower mechanical strength and considerably higher CGC value (>0.05 wt%) compared to Fmoc-Lys (Fmoc)-Asp.

It is worth mentioning that not all the Fmoc-modified dipeptides form hydrogels in specific conditions, and most of those dipeptides generally form hydrogels at low pH, which limited the applications in biomedical eras (Fichman and Gazit, 2014; Hendi et al., 2020; Zhang and Huang, 2021). Inspired by the success of Fmoc-modified dipeptide hydrogelators, various aromatic moieties, such as phenyl, naphthalene (Nap), azobenzene, and pyrene derivatives, have been utilized to augment the hydrogelation or to introduce functionality at the N-terminus of dipeptide (Fleming and Ulijn, 2014). Among the N-terminal aromatic-modified dipeptide hydrogelators, Nap-based dipeptides have been the most widely studied. Nap-modified dipeptides can usually form hydrogels, just like Fmoc-modified dipeptides, by adjusting the solution pH. Adams and coworkers have demonstrated that the gradual removal of the charge allows lateral assembly of the molecules to form fibrous structures by π–π stacking and β-sheet formation (Chen et al., 2010a). They have also found out that the nitrile or bromo groups substituted Nap-modified dipeptide have shown better hydrogelation properties than non-substituted ones, suggesting that the electron-withdrawing nature of the bromo and nitro groups, which reduced the electron density of the π-system, has an impact on the self-assembly properties of the dipeptides *via* aromatic stacking interactions. Moreover, Nap-Gly-Ala and Nap-Ala-Gly self-assemble to different results as the pH of solution dropped: Nap-Gly-Ala is induced to hydrogel, but Nap-Ala-Gly is induced to crystallization (Chen et al., 2010a; Adams et al., 2010).

A Nap-based dipeptide 2Nap-Phe-Phe can pack into hollow tubes in the micellar state at high pH (McAulay et al., 2019), and when the pH decrease, gels are formed because the hollow core is lost, and lateral association leads to elliptical structures that entangle to form the gels (Draper et al., 2020). Furthermore, the micellar aggregates formed at high pH for dipeptide-based gelators can be varied by simply using different salts to raise the pH (McAulay et al., 2020). The change in cations leads to different packing in the micellar phase and the formation of different structures, thus affecting the properties of the resulting gels.

Besides Fmoc and Nap groups, several other aromatic groups have also been researched in N-terminal dipeptide modifications. Gazit and coworkers have shown that N-terminal carboxybenzyl (Cbz)-modified dipeptide Phe-Phe could form hydrogels with different structures (such as nanowires, fibers, nanospheres, and nanotoroids) by changing the starting solvent (Brown et al., 2018). A library of Cbz-modified dehydrodipeptides Cbz-L-Xaa-Z-ΔPhe-OH (Xaa = Met, Phe, Tyr, Ala, Gly) were synthesized for hydrogel screening by Ferreira et al. (Veloso et al., 2021). The Cbz-L-Met-Z-ΔPhe-OH and Cbz-L-Phe-Z-ΔPhe-OH could form self-assembly hydrogels and could be used as drug carriers for the delivery of curcumin and doxorubicin.

**FIGURE 3:**
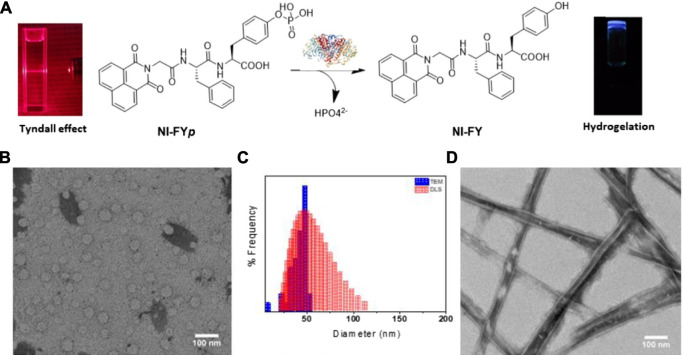
**(A)** The illustration of self-assembly NI-FY*p* dipeptide nanostructures which transformed from spherical nanoparticles into a hydrogel during the enzymatic catalytic process; **(B)** TEM image of NI-FY*p* nanoparticles in Tris-buffer (10 mM); **(C)** particle size distribution histogram of NI-FY*p* (10 mM) nanoparticles by TEM and DLS measurements; **(D)** TEM images of NI-FY*p* treated with 10 U of ALP (Chakravarthy et al., 2020).

In recent years, naphthalimide (NI) derivatives have received increasing attention because of their unique photophysical property and photostability (Schab-Balcerzak et al., 2015). Lin group have reported four 1,8-naphthalimide (NI)-modified dipeptide hydrogelators and found out that NI-Phe-Tyr, NI-Tyr-Phe, and NI-Tyr-Tyr could form hydrogels under physiological pH conditions (Hsu et al., 2018). Furthermore, the NI-Tyr-Phe showed low cytotoxicity and could be applied as a suitable carrier for drug delivery. They have also developed NI-modified phosphate-based dipeptide NI-FY*p* (Chakravarthy et al., 2020) (Figure 3), which formed spherical nanoparticles in the aqueous condition firstly, and then in the condition of enzymatic catalysis, they slowly transformed into partially unzipped nanotubular structures and subsequently resulted in hydrogelation.

2-Thiophene-modified diphenylalanine was reported by Draper et al. that it could form transparent hydrogel from the initially turbid gel after 3 days due to the effect of carbon dioxide, which implied that the molecular rearrangement of the gelator molecules and the self-assembly structures were becoming smaller or thinner (Draper et al., 2015).

**FIGURE 4:**
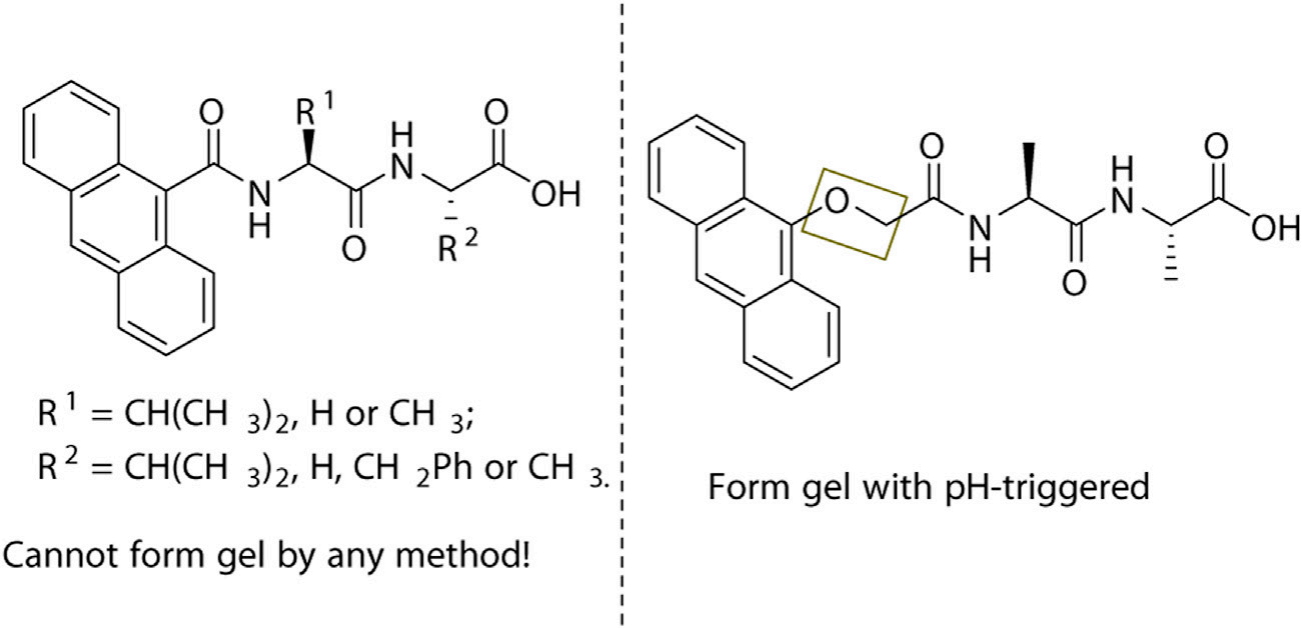
The linker between the anthracene ring and dipeptide can affect gel formation.

An anthracene ring is an interesting group in the dipeptide hydrogelators researches. When the anthracene was directly linked to the N-terminal of dipeptide by amide and no other group between amide and anthracene, no formed hydrogels were discovered, while a similar structure, but with an additional OCH_2_ spacer between the anthracene ring and first amide bond, was an effective hydrogelator (Chen et al., 2010b; Awhida et al., 2015) (Figure 4). However, pyrene and Nap-modified dipeptide, whether those conjugated directly or with a longer spacer, were all reported to be effective hydrogelators.

**FIGURE 5:**
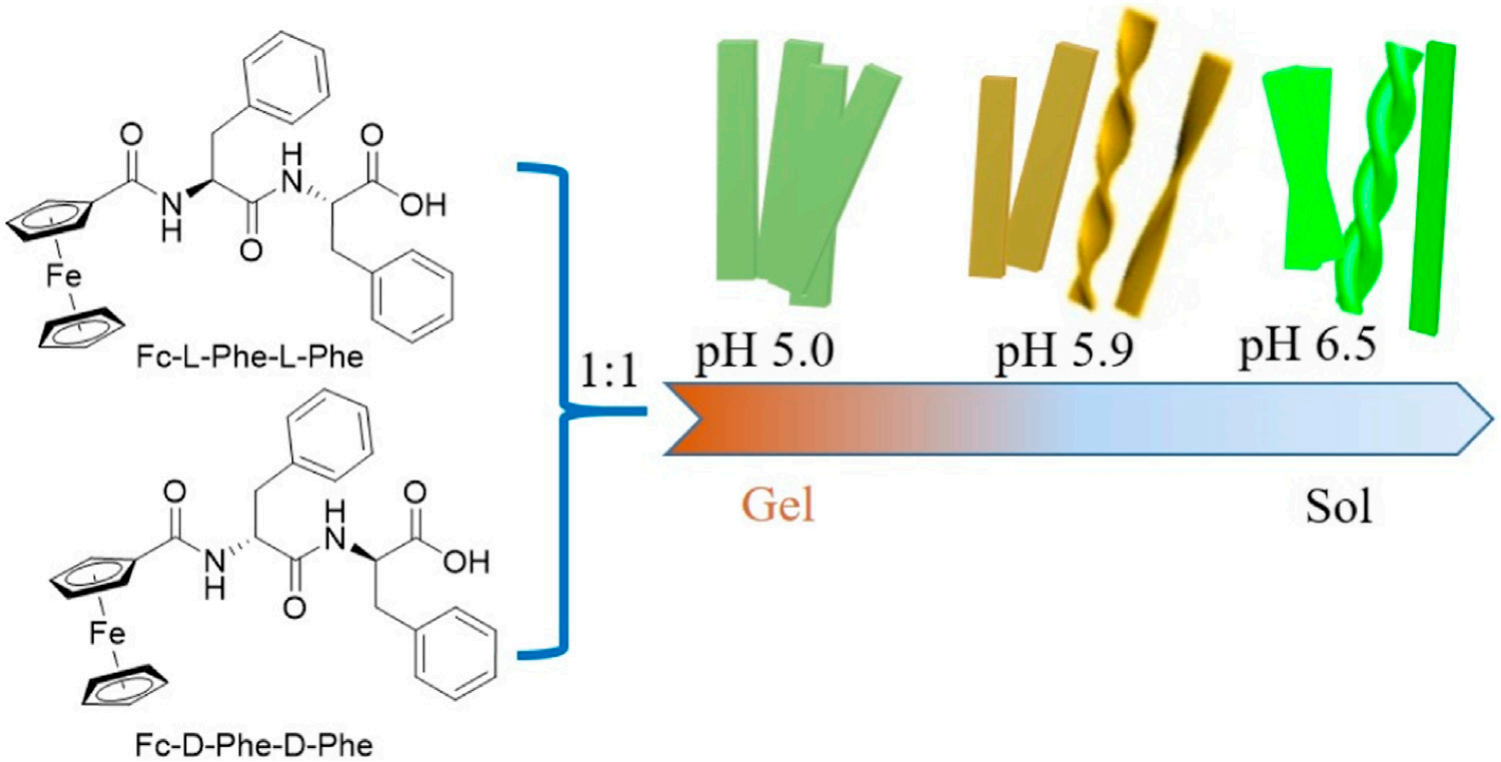
The molecular structure of the Fc-L-Phe-L-Phe-OH and Fc-L-Phe-L-Phe-OH; the illustration of the pH responsiveness of the hydrogel (Zhang et al., 2020).

Due to the redox activity of ferrocene (Fc), the Fc-modified peptides have attracted great interest in biomedicine and sensing. Qi et al. have reported that the Fc-Phe-Phe could form a self-supporting hydrogel under kinetic control by introducing a mechanical force, which showed a morphological transition from metastable nanospheres to nanofibers. The strong hydrophobic interaction of the Fc moiety was suggested to have a key role in this kinetically controlled self-assembly process. Moreover, Wang and coworkers have reported that the same Fc-modified dipeptide Fc-Phe-Phe could self-assemble into an ultra-pH-sensitive chiral hydrogel, which formed at a very narrow pH range of 5.7–5.9 (Zhang et al., 2020). The pure Fc-L-Phe-L-Phe or Fc-D-Phe-D-Phe peptide could form self-assemble into right- or left-handed nanohelices in a mixture of water and organic solvent, which indicated that molecular chirality had a great influence on the gelation of the peptides and water molecules are essential in directing the chiral self-assembly of Fc-Phe-Phe into entangled chiral nanostructures, leading to the formation of stimuli-responsive chiral hydrogels (Figure 5).

**FIGURE 6:**
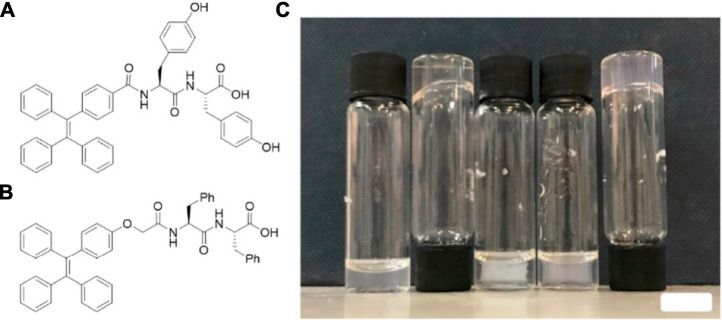
The structure of **(A)** TPE-Tyr-Tyr reported by Lin group (Yeh et al., 2016); **(B)** TPE-Phe-Phe (TPE-FF) reported by Adams (Castilla et al., 2018); **(C)** from left to right, a solution of TPE-FF at 5 mg/ml at high pH, at 10 mg/ml after a heat-cool cycle (formed hydrogel), at 5 mg/ml after the addition of glucono-dlactone (GdL), at 5 mg/ml after the addition of CaCl_2_, and at 5 mg/ml after the addition of NaCl (18 mg/ml) (formed hydrogel) (Castilla et al., 2018).

Tetraphenylethylene (TPE), a supramolecular fluorescent material (SFM) with aggregation-induced emission characteristics, has also been reported with dipeptides as hydrogelators. Lin and his coworkers have found out that the TPE-modified dipeptide TPE-Gly-Gly could be a hydrogelator under basic media (pH = 10.5) and failed to form hydrogels at neutral pH conditions, which are considered as the main drawback for biomaterials (Yeh et al., 2016). Their subsequent studies about TPE-modified dipeptides have indicated that TPE-Phe-Phe was too hydrophobic, insoluble in any aqueous conditions, and thus difficult to form a hydrogel, while TPE-Tyr-Tyr (Figure 6A) was able to form hydrogel under a broad pH range from 3.7 to 10.2 (Talloj et al., 2020). The hydrogel formed by TPE-Tyr-Tyr showed tunable morphology *via* pH: as the pH of the hydrogel decreases, the nanostructure transformed from nanofiber to flat nanobelts and then to twisted nanobelts. Furthermore, the TPE-Tyr-Tyr displayed selective cell adhesion response for 3A6 cells (Human MSCs), which have potential applications in tissue engineering such as stem cell-based therapies.

Adams et al. have described other TPE-base dipeptides, while the TPE was linked to dipeptide *via* 2-[4-(1,2,2-triphenylethenyl)phenoxy]acetic acid (Figure 6B) (Castilla et al., 2018). Their hydrogel formed by TPE-Phe-Phe showed syneresis properties, and further studies have indicated that the molecular packing in the calcium chloride and sodium chloride-triggered gels is different (Figure 6C). Under high pH conditions, the TPE-Phe-Phe solution initially could form a gel after adding calcium chloride solution but dehydrated again over time, which suggested that the samples were not self-supporting. On the contrary, the addition of sodium chloride could lead to the formation of a hydrogel. This suggested that the linker between the TPE and peptide component is also important to the properties of the hydrogels. The linker of TPE in Adams’ research has more flexibility than that in Lin’s researches (Yeh et al., 2016), which means that Adams’s TPE is not highly restricted in molecular packing and the corresponding hydrogels may have different properties.

### Non-Aromatic-Modified Dipeptide

Although N-terminal aromatic-modified dipeptides have shown interesting gelation properties in relation to stabilities and mechanicals, it should be noted that the literature has reported the toxicity of those groups, which may limit the biomedical applications of those gels. For example, degradation products of Fmoc-Phe-Phe showed some cytotoxicity because the highly reactive dibenzofulvalene was formed upon cleavage of the Fmoc group (Truong et al., 2015; D.; Martin and Thordarson, 2020). Naproxen-capped dipeptide Nap-Phe-Phe only showed biocompatibility at low concentrations and the IC_50_ value is lower than the minimum gel concentration, which suggested that the molecule was not suitable for biomedical applications (Li et al., 2013).

**FIGURE 7:**
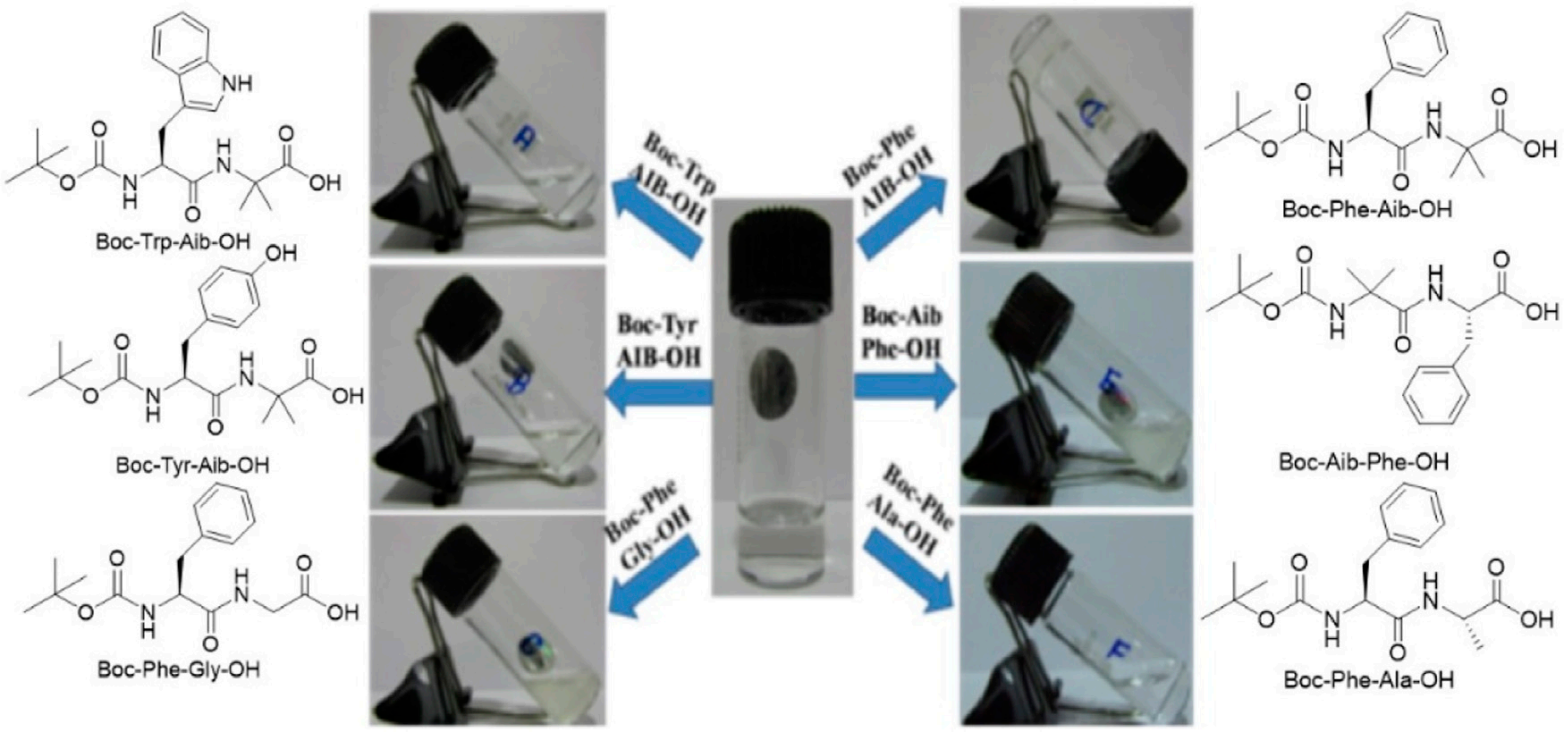
The structures of dipeptide Boc-Phe-Aib analogs and their gelation studies (Nandi et al., 2018).

Haldar et al. have reported a N-(tert-butoxycarbonyl) (N-Boc)-modified dipeptide Boc-Phe-Aib (Nandi et al., 2018), which transformed into a robust hydrogel upon addition with three equivalents of sodium hydroxide and water (Figure 7). The other similar analogs of dipeptides, such as Boc-Tyr-Aib, Boc-Trp-Aib, Boc-Phe-Ala (Sangeetha and Maitra, 2005), Boc-Phe-Gly (O’Leary et al., 2011), and Boc-Aib-Phe, have all failed to form such a hydrogel under the same condition. Haldar and the coworkers have found out that the N-Boc-protected dipeptide Boc-Phe-Val could form a hydrogel in the mixtures of NH_4_OH and NaCl; Na_2_CO_3_ and LiOH; and NaCl and KOH (Podder et al., 2019). This result suggested that sodium and hydroxide ions both played a key role in hydrogel formation.

**FIGURE 8:**
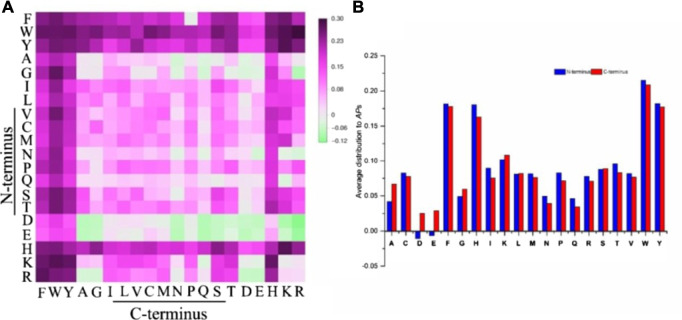
**(A)***AP*s scoring distribution of all 400 C13-dipeptides after 50ns CG-MD simulations. **(B)** Average distribution to *AP*s of specific amino acids in the N-terminus (blue) and C-terminal position (red) (Hu T. et al., 2020).

Recently, self-assembly hydrocarbonyl chain-modified dipeptides (C_n_-dipeptides) with lower toxicity and greater biocompatibility than those modified with Boc, Nap, and/or Fmoc have attracted more and more attention. The Adams group has studied the hydrogelation properties of various carbon chain lengths for modified dipeptide C_n_-Phe-Phe and found out that C_12_-CO-(C_13_-) was the optimal group for dipeptide hydrogelators (Chen et al., 2013). In order to understand the effects of the introduction of C_13_- into the dipeptides, the combined computational and experimental methods were used to investigate the self-assemblies and gelations of C_13_-dipeptides (Hu T. et al., 2020). They have tried to introduce a modified parameter-aggregation propensity (AP_S_), which accounted for side chain effects, to help design peptide-based gelators (Figure 8). From those experimental results, the AP_S_ values successfully demonstrated the tendency of self-assembly of the C_13_-dipeptides even though the models were based on a neutral environment. However, the drawback of the selected C_13_-dipeptides is that the hydrogels are usually formed at low pH (∼4.0), which would limit the applications of those hydrogels. Banerjee and coworkers (Baral et al., 2015) have also reported that a C_12_-modified dipeptide (C_12_-Ala-Ala) could form a hydrogel in phosphate buffer in the pH range of 7.0–8.5, whereas the natural dipeptide (Ala-Ala) could not form a gel.

## C-Terminal Modified Dipeptides

The C-terminal carboxylic acid moieties of dipeptides could usually be modified to perturb the hydrophobicity and hydrogen bond capacity of the C-termius, which showed significant effects on the self-assembling propensity, such as the hydrogelation ability and morphology of the self-assembled nanostructures.

**FIGURE 9:**
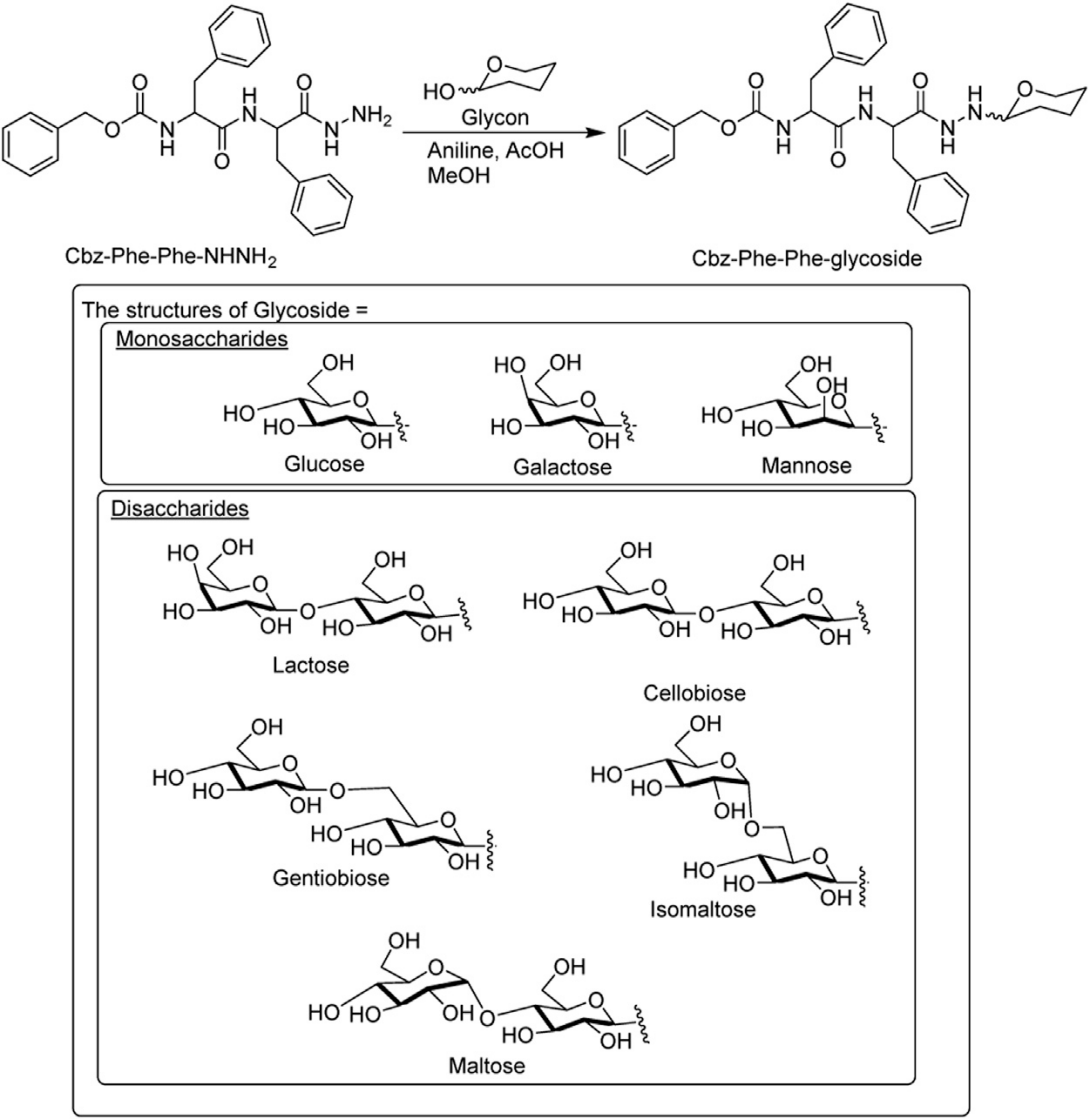
The structures of C-terminal modified dipeptides by Ikeda et al.

Tuttle and Ulijn et al. have provided a combined experimental/computational method to evaluate the hydrogelation properties of various C-terminus modifications (amide vs. ester) of Fmoc-dipeptide (Sasselli et al., 2016). The approach proved that amide modification was more important than ester modification because the Fmoc-Thr-Phe-NH_2_ could form more stable hydrogels than Fmoc-Thr-Phe-OMe, possibly due to the extra hydrogen bonds that the amide group forms upon self-assembly. Ikeda et al. have reported C-terminal hydrazide-modified dipeptide, Cbz-Phe-Phe-NHNH_2_, which showed limited aqueous solubility and could not form a hydrogel, but its carbohydrate derivatives could form a hydrogel (Tsuzuki et al., 2017) (Figure 9). They have found out that the disaccharide structures (epimer or glycosidic-bond geometry) have a significant effect on the formation ability of the hydrogel and the morphology of the self-assembled structures. The hydrazide group C-terminal modified p-nitro-phenylmethoxycarbonyl (NPmoc)-based dipeptide NPmoc-Phe-Ala-NHNH_2_ could form a hydrogel, while the others like NPmoc-Ala-Phe-NHNH_2_, NPmoc-Phe-Phe-NHNH_2,_ and NPmoc-Ala-Ala-NHNH_2_ failed to form hydrogel at the same conditions (Ohtomi et al., 2020). Their results also indicated that adjusting the side chain phenyl group position in the dipeptide hydrazides could vary the dimensions of the self-assembled nanostructures from 1D (fiber and tubular) to 2D (sheet).

**FIGURE 10:**
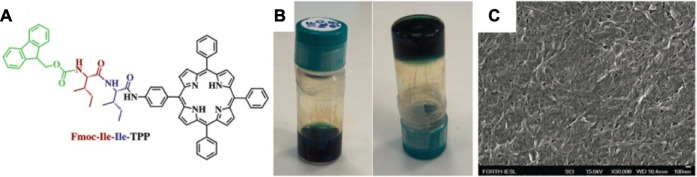
**(A)** The structure of Fmoc-Ile-Ile-TPP; **(B)** photographs of the hydrogel formed from the Fmoc-Ile-Ile-TPP in HFIP/H_2_O (2/8, v/v, 1 mM); **(C)** FESEM image of the formed hydrogel (Nikoloudakis et al., 2019).

Coutsolelos et al. have synthesized several aliphatic dipeptides bearing various protecting groups and found out that Fmoc-Ile-Ile-TPP (TPP: tetraphenyl porphyrin) formed a hydrogel in HFIP–water (2:8) solvent mixture, whereas TPP-Ile-Ile-OMe and Boc-Ile-Ile-TPP failed to form hydrogel due to the spherical assemblies in solvents (Figure 10) (Nikoloudakis et al., 2019).

## Uncapped Dipeptide

Although uncapped dipeptides are very attractive building blocks due to their chemical simplicity, they usually fail to form a stable hydrogel, e.g., the Phe-Phe hydrogels were reported to be metastable (Conte et al., 2016; Kurbasic et al., 2019; Kralj et al., 2020), unless the Phe benzene ring of the dipeptide was further modified by adding a p-nitro (Kurbasic et al., 2019) or p-iondin (Kralj et al., 2020) substitution. For the uncapped dipeptide composed of natural amino acids, Ventura and coworkers have reported that Ile-Phe dipeptide can form a stable hydrogel at pH 5.8 (de Groot et al., 2007); later, Marchesan et al. found out that Leu-Phe dipeptide could form a stable hydrogel in phosphate buffer (Bellotto et al., 2020). However, besides these two dipeptides, no other dipeptide hydrogelator composed of natural amino acids has been reported to form a stable hydrogel.

**FIGURE 11:**
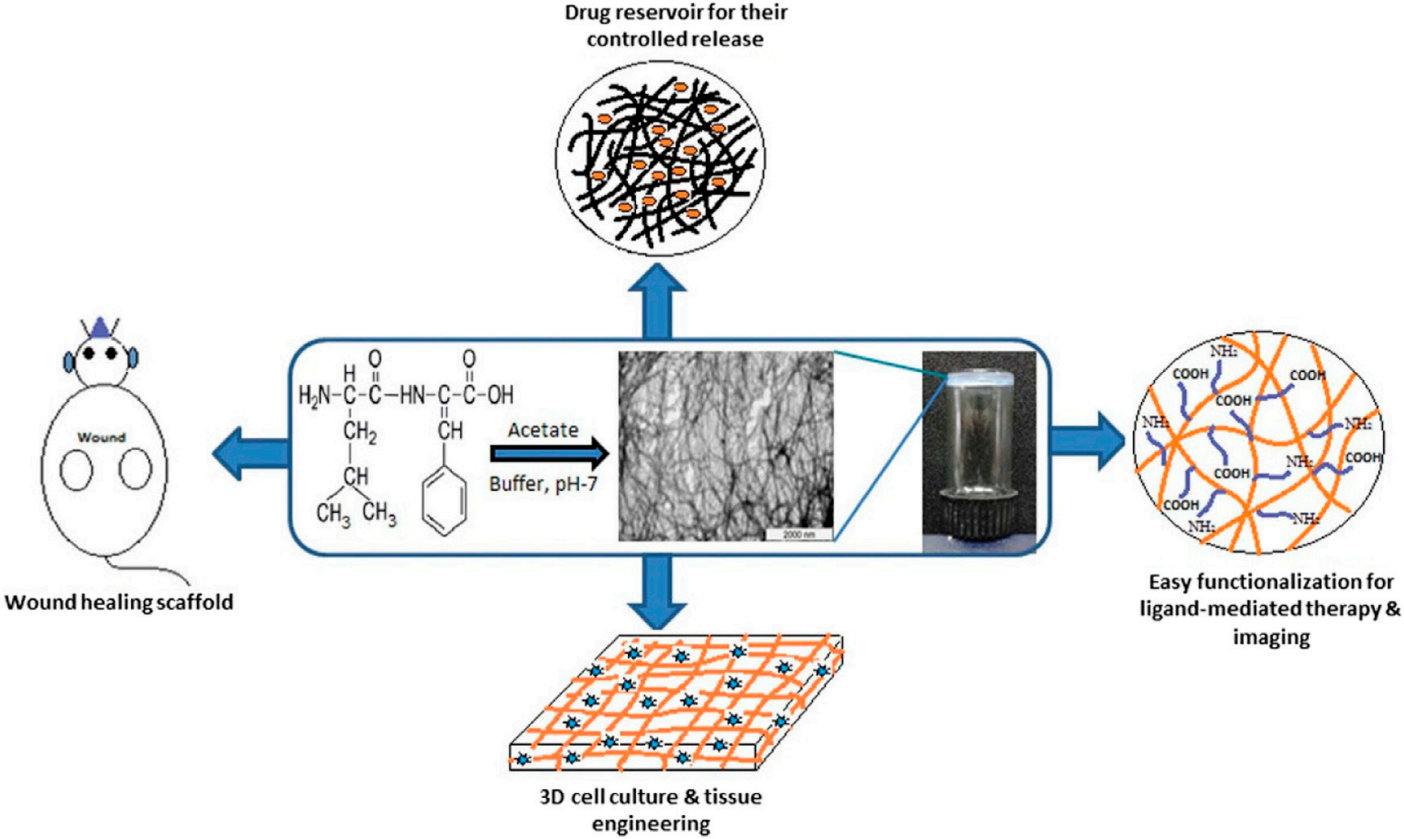
LeuΔPhe can be self-assembled into a stable hydrogel at room temperature and applied in the biomedical field (Thota et al., 2016).

α,β-Dehydrophenylalanine (ΔPhe), an unsaturated analog of Phe, was found to induce conformational constraints in the peptide backbone and to usually yield a hydrogel. Chauhan et al. have reported that uncapped dipeptide Phe-ΔPhe could self-assemble into a stable hydrogel at minimal gelation concentrations of 0.2 wt% in a buffer solution at pH 7.0 (Panda et al., 2008). Yadav and Thota have reported another ΔPhe-containing dipeptide, Leu-ΔPhe, which could form a highly stable and mechanically strong hydrogel under mild physiological aqueous conditions (Thota et al., 2016). These hydrogels from ΔPhe-containing dipeptide were found to be nontoxic and were used to entrap several hydrophobic and hydrophilic drug molecules and release them in a controlled manner (Figure 11).

**FIGURE 12:**
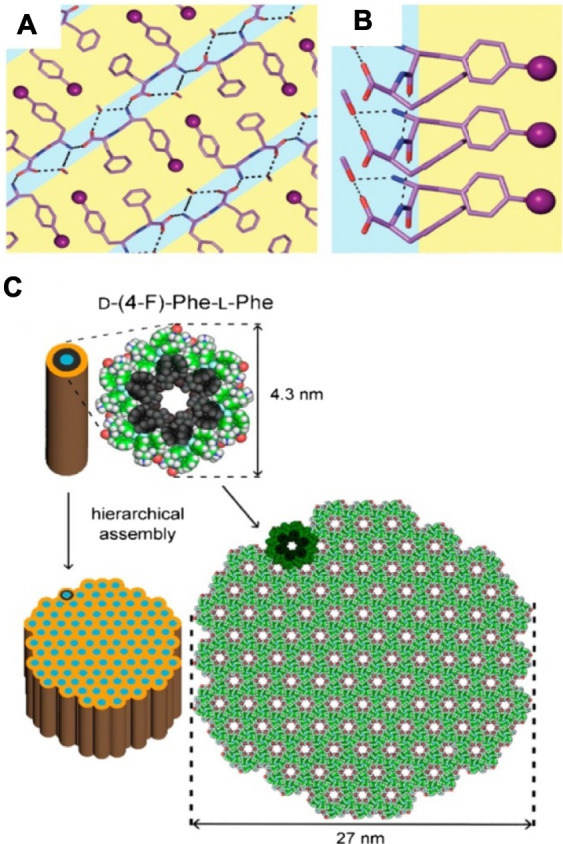
**(A)** and **(B)** Single-crystal XRD structure of D-(4-I)-Phe-L-Phe (CCDC 016374). The light blue represents the hydrophilic layers and iodine atoms (purple sphere) allowed Phe side chains in hydrophobic layers (yellow); **(C)** single-crystal XRD structure (CCDC 2016373) of D-(4-F)-Phe-L-Phe, which can pack to an intermediate level of bundling to generate 27 nm wide fibrils as observed by TEM (Kralj et al., 2020).

The chirality of the composed amino acids affects the hydrogelation ability of the uncapped dipeptide. Marchesan et al. chose diphenylalanine as a model compound and studied the self-organization of heterochiral D-Phe-L-Phe and its halogenated derivatives (Figure 12, Kralj et al., 2020). Their studies showed that D-Phe-L-Phe self-assembled into nanofibrils and resulted in a transparent hydrogel. Besides, they also found that halogenation had significant effects on the self-assembling process. For example, fluorination induced the analogous packing to nanotube formation, and iodination was the most effective strategy to augment the stability of the hydrogel.

## Multicomponent Self-Assembly

**FIGURE 13:**
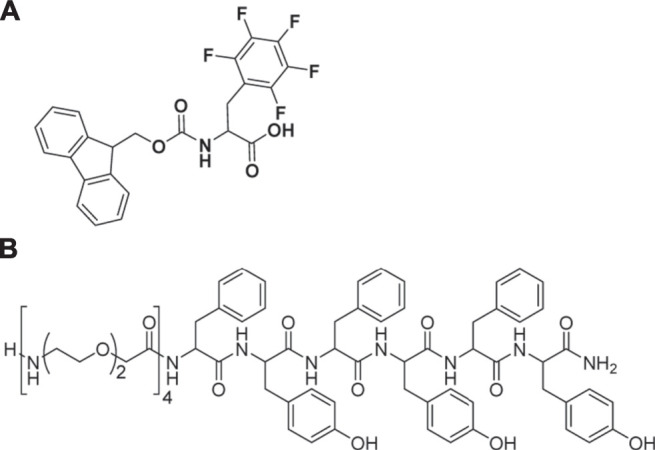
**(A)** The structure of pentafluorinated Fmoc-Phe used by Halperin-Sternfeld et al. (Halperin-Sternfeld et al., 2017); **(B)** The structure of PEGylated (Phe-Tyf)_3_ hexapeptide.

Multicomponent self-assembly is a hot topic at present, which generally has advantages over a single component system (Li et al., 2021a). Multicomponent self-assembly of two or more different building blocks into one ordered nanostructure can promote the formation of a wider and more complex architecture, provide the tunable mechanical properties, enhance stability, and provide spatiotemporal control of self-assembly (Raeburn and Adams, 2015; Halperin-Sternfeld et al., 2017; Makam and Gazit, 2018). Recently, multicomponent hydrogels containing dipeptides have also attracted interest due to the innovative scaffolds from multicomponent self-assembly. Some studies have reported that the multicomponent self-assembly containing dipeptides has significant effects on the mechanical properties of those formed hydrogels. For example, Tendler and coworkers have reported that the mechanical properties of hydrogels formed by two peptides Phe-Phe and di-D-2-napthylalanine could be fine-tuned *via* changing their relative concentration (Sedman et al., 2013). Abramovich et al. have analyzed the gelation kinetics of mixed Fmoc-Phe-Phe/pentafluorinated Fmoc-Phe (Figure 13A) and discovered that the mechanical properties were improved (Halperin-Sternfeld et al., 2017). In the same way, the mix of Fmoc-Phe-Phe and Fmoc-Arg can form new hydrogels, which demonstrated high mechanical strength with a storage modulus of up to 29 kPa in combination with the bone mineral hydroxyapatite (Ghosh et al., 2017).

Fmoc-Phe-Phe and hexapeptide-based multicomponent hydrogels were studied by the Accardo group, and they found out that the combination of Fmoc-Phe-Phe with (Phe-Tyf)_3_ hexapeptide or its PEGylated analog (Figure 13B) at different volumetric ratios (2/1 or 1/1 v/v) could provide hydrogels with tunable mechanical properties (Diaferia et al., 2019a). For instance, the PEGylated gel showed a decrease of the gel rigidity and slowing down of the gel formation kinetics compared to the un-PEGylated gel, and thus the mixed hydrogel Fmoc-Phe-Phe/PEG_8_-(Phe-Tyf)_3_ (1/1) have minor rigidity compared to Fmoc-Phe-Phe/PEG_8_-(Phe-Tyf)_3_ (2/1).

**FIGURE 14:**
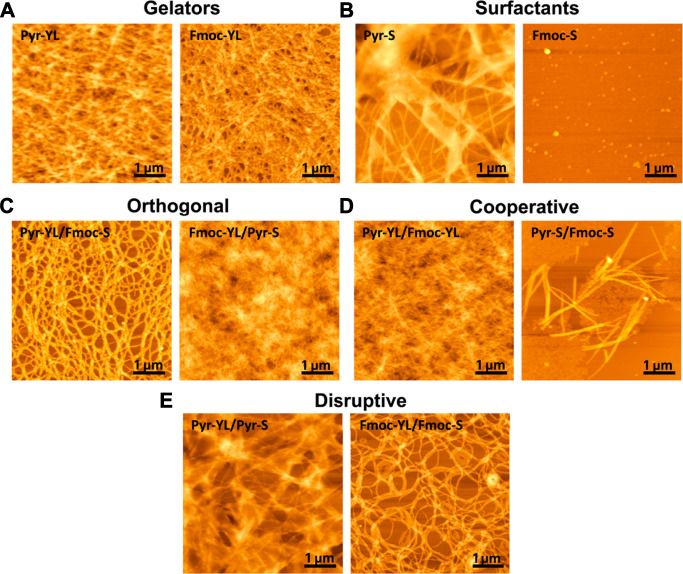
AFM of **(A)** the gelators Pyr-YL and Fmoc-YL; **(B)** the surfactants Fmoc-S and Pyr-S; **(C)** orthogonal Pyr-YL/Fmoc-S and Fmoc-YL/Pyr-S; **(D)** cooperative Pyr-YL/Fmoc-YL and Pyr-S/Fmoc-S; **(E)** disruptive Pyr-YL/Pyr-S and Fmoc-YL/Fmoc-S (Fleming et al., 2014).

Ulijn and coworkers examined four different combinations of Fmoc- and pyrene (Py)-modified compounds (Fleming et al., 2014). The results showed that the combinations of two structurally different peptides (Py-Tyr-Leu/Fmoc-Ser and Fmoc-Tyr-Leu/Py-Ser) resulted in orthogonal coassembly, while the structurally similar peptides (Py-Tyr-Leu/Fmoc-Tyr-Leu and Py-Ser/Fmoc-Ser) followed the cooperative coassembly process (Figures 14A,B,D). They discovered that Pyr-Ser could perturb the intermolecular H-bonding and fiber formation associated with Fmoc-Tyr-Leu and Py-Tyr-Leu (Figures 14C,E), while Fmoc-Ser showed a disruptive effect on the self-assembly structure of Fmoc-Tyr-Leu (Figure 14E).

Yan et al. utilized the combination of dipeptide Fmoc-Phe-Phe and fullerene to improve the mechanical properties of the hydrogel for a better injectable formulation for biomedical applications (Zhang et al., 2018). Due to the coassembly of dipeptide and fullerene, the aggregation of fullerene in the hydrogel was largely inhibited *via* the noncovalent interactions between the dipeptide and the fullerene, and at the same time, fullerene enhanced the mechanical properties of the dipeptide hydrogel. The incorporation of the fullerene profoundly improved the photodynamic therapy (PDT) efficiency compared to the untreated fullerene, and the dipeptide-fullerene hydrogels could effectively inhibit multiantibiotic-resistant *Staphylococcus aureus* both *in vitro* and *in vivo*.

## Conclusion

Self-assembly dipeptide hydrogel is the result of coordinated noncovalent interactions among molecular, environmental, and kinetic considerations. Although some successful dipeptide hydrogels have been reported, it is still difficult to predict and control the results of molecular assembly, which are derived by various factors that are external (solvent, temperature, light, etc.) and internal (molecular geometry, hydrophobicity, electronics, etc.). Nonetheless, through the subtle design of dipeptide molecules and the modification of the N- and C-terminus, dipeptide hydrogels can have great potential applications in preparing minimal biocompatible materials.
